# Comparison of TRIBE and STAMP for identifying targets of RNA binding proteins in human and *Drosophila* cells

**DOI:** 10.1261/rna.079608.123

**Published:** 2023-08

**Authors:** Katharine C. Abruzzi, Corrie Ratner, Michael Rosbash

**Affiliations:** Howard Hughes Medical Institute, Brandeis University, Waltham, Massachusetts 02454, USA

**Keywords:** RNA binding proteins, ADAR, APOBEC

## Abstract

RNA binding proteins (RBPs) perform a myriad of functions and are implicated in numerous neurological diseases. To identify the targets of RBPs in small numbers of cells, we developed TRIBE, in which the catalytic domain of the RNA editing enzyme ADAR (ADARcd) is fused to an RBP. When the RBP binds to an mRNA, ADAR catalyzes A to G modifications in the target mRNA that can be easily identified in standard RNA sequencing. In STAMP, the concept is the same except the ADARcd is replaced by the RNA editing enzyme APOBEC. Here we compared TRIBE and STAMP side-by-side in human and *Drosophila* cells. The goal is to learn the pros and cons of each method so that researchers can choose the method best suited to their RBP and system. In human cells, TRIBE and STAMP were performed using the RBP TDP-43. Although they both identified TDP-43 target mRNAs, combining the two methods more successfully identified high-confidence targets. In *Drosophila* cells, RBP–APOBEC fusions generated only low numbers of editing sites, comparable to the level of control editing. This was true for two different RBPs, Hrp48 and Thor (*Drosophila* EIF4E-BP), indicating that STAMP does not work well in *Drosophila*.

## INTRODUCTION

RNAs are bound by RNA binding proteins (RBPs), even in the nucleus before transcription is complete. For example, the association of RBPs with pre-mRNA affects all aspects of nuclear RNA processing, from capping, splicing, and 3′-end formation to nuclear export (for review, see Hocine et al. 2010). Additional RBPs bind to cytoplasmic mRNAs and determine their subcellular localization and stability (Das et al. 2021). Yet, other RBPs act to regulate translation including the association of specific mRNAs with the ribosome (Babitzke et al. 2009). The coordinated functioning of these RBPs is necessary to generate the right amount of the correct protein, at the right time and in the right place.

Many human neurological diseases are linked to RBP mutations. This vulnerability may be due in part to the fact that most central brain neurons are long-lived and cannot divide themselves out of trouble. For example, mutations in FMRP are responsible for Fragile X syndrome (Penagarikano et al. 2007), dysregulation of TDP-43 underlies amyotrophic lateral sclerosis (ALS; Ling et al. 2013; Gao et al. 2019), and mutations in SMN1 cause spinal muscular atrophy (SMA; Farrar and Kiernan 2015). It is therefore important to understand the mRNA targets of key neuronal RBPs.

For many years, crosslinking immunoprecipitation (CLIP) has been the tried-and-true method for identifying the targets of RBPs. CLIP is a powerful method because UV crosslinking is used to biochemically attach the RBP to the target mRNA, which makes precise binding site identification possible. However, CLIP also has drawbacks: an efficient antibody is needed, crosslinking is biased to guanosine and thymidine residues, crosslinking can capture weak or transient interactions, and a very large amount of biological material is usually required (for review, see Hafner et al. 2021; Xu et al. 2022). As we began to understand more about the heterogeneity of tissues and even single cells, it became apparent that a method that can identify RBP targets in small amounts of material was needed.

In the past seven years, two methods have been developed that allow researchers to examine the targets of RBP in small discrete groups of cells. The first was from our laboratory in 2016 and is called targets of RNA binding proteins identified by editing (TRIBE; McMahon et al. 2016). In TRIBE, an RBP of interest is fused to the catalytic domain of ADAR (ADARcd). When the RBP binds its target mRNA, the ADARcd edits the mRNA. In 2018, we incorporated a single point mutation in the ADARcd, which substantially increased editing activity and decreased local sequence preferences (Kuttan and Bass 2012); this variant has been used for all recent TRIBE experiments (HyperTRIBE; Xu et al. 2018). Editing sites are then identified computationally from RNA sequencing data. Importantly, this allows target identification in very small numbers of cells and neurons without requiring biochemistry. TRIBE has been useful to a number of other labs working in different fields and model organisms, ranging from humans to malaria parasites (Liu et al. 2019; Alizzi et al. 2020; Nguyen et al. 2020; Arribas-Hernández et al. 2021; Cheng et al. 2021; Singh et al. 2021; van Leeuwen et al. 2022).

A very similar strategy, conceptually and in overall design, was recently published in which an RBP is fused to a different editing enzyme, APOBEC1 (apolipoprotein B mRNA editing enzyme, catalytic polypeptide-like). This method was used in two different studies and named STAMP (Brannan et al. 2021) and Dart-seq (Meyer 2019); we will use the nomenclature STAMP in this paper. TRIBE and STAMP both offer distinct advantages and disadvantages. ADAR is an adenosine deaminase and makes A-to-I edits within a supposedly double-stranded region even without the RNA binding regions normally present in ADAR (Macbeth et al. 2005). APOBEC1 is a cytidine deaminase and therefore catalyzes C-to-T edits in single-stranded RNA but also edits single-stranded DNA (Rosenberg et al. 2011; Smith et al. 2012; Salter et al. 2016). ADAR and APOBEC both have some local sequence preferences (UAG → ADAR; A/U flanked → APOBEC; Rosenberg et al. 2011; Kuttan and Bass 2012). TRIBE only fuses the ADARcd, which can be cleanly separated from the ADAR double-stranded RNA binding regions to the RBP. The latter normally directs the specificity of full-length ADAR and is replaced by the RBP in TRIBE. In contrast, the entire APOBEC protein is fused in STAMP, perhaps because it is uncertain how the editing specificity of APOBEC is defined. For example, APOBEC-mediated editing may require dimerization as well as cofactors (e.g., A1CF and RBM47; for review, see Smith et al. 2012; Fossat et al. 2014).

Given the similarity of TRIBE and STAMP, it is perhaps uncertain which method one should choose to identify targets of an RBP of interest. We therefore examined these methods side-by-side in HEK-293 and *Drosophila* cells to identify the pros and cons of each method experimentally. In HEK cells, the human ADAR2cd (referred to in this manuscript as ADAR; 44 kDa) and rat APOBEC1 (referred to in this manuscript as APOBEC; 25 kDa) were fused to Tar DNA binding protein-43 (TDP-43). We previously successfully used this protein for TRIBE in a mammalian system (Herzog et al. 2020).

As indicated by published STAMP results (Brannan et al. 2021), APOBEC worked well in HEK cells. TDP-43-APOBEC generated a similar number of editing sites as TDP-43-ADAR, >10-fold more sites than expressing the editing enzymes alone. TDP-43-ADAR and TDP-43-APOBEC both identified substantial numbers of target genes, although TDP-43-ADAR identified 50% more genes than TDP-43-APOBEC. Moreover, 70% of the TDP-43-APOBEC target genes were also identified by TDP-43-ADAR. We also compared STAMP and TRIBE in *Drosophila*, with two RBPs, Hrp48, and Thor (*Drosophila* EIF4E-BP). Targets of both proteins had been successfully identified with TRIBE (McMahon et al. 2016; Jin et al. 2020). To test STAMP in *Drosophila*, we replaced the ADARcd with APOBEC to generate Hrp48 and Thor STAMP. Although TRIBE worked well with both of these RBPs, as expected there was no substantial editing of target mRNAs with STAMP.

## RESULTS

**FIGURE 1.**
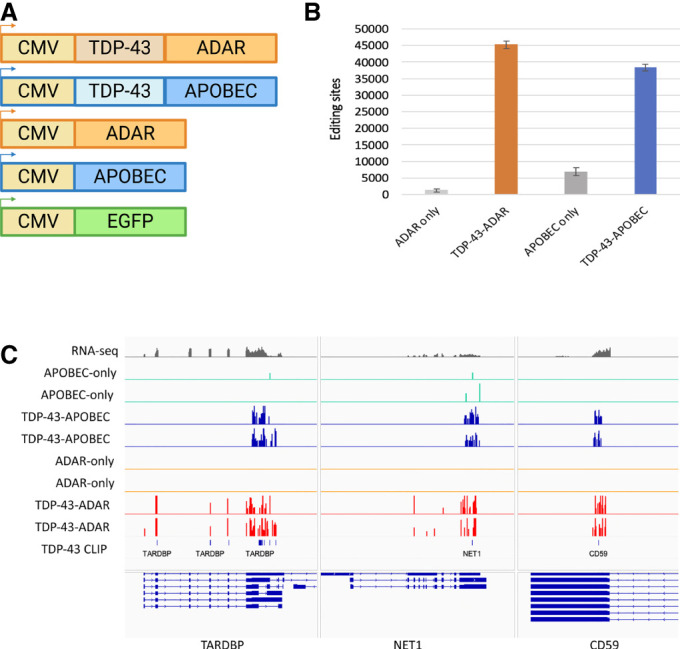
Both TDP-43 TRIBE (ADAR) and TDP-43 STAMP (APOBEC) identify candidate TDP-43 targets in HEK-293 cells. (*A*) Design of constructs for the expression of TDP-43-ADAR, TDP-43-APOBEC and their respective controls in HEK-293 cells. All transgenes were expressed using the cytomegalovirus (CMV) promoter. (*B*) The average number of editing sites identified in HEK-293 cells expressing TDP-43-ADAR, TDP-43-APOBEC, ADAR, or APOBEC alone. The graph quantifies A to G changes for ADAR and C to T changes for APOBEC. Two biological replicates are shown. Error bars indicate standard deviation. (*C*) Visualization of RNA editing sites generated by TDP-43-ADAR (red) and TDP-43-APOBEC (blue) on TARDBP (the gene encoding TDP-43), NET1 and CD59. The Integrated Genomics Viewer (IGV; Robinson et al. 2011; Thorvaldsdottir et al. 2013) visualization of two biological replicates of TDP-43-ADAR (red), TDP-43-APOBEC (blue), ADAR alone (orange), and APOBEC alone (green). *Y*-axis is 20% for all editing site tracks. RNA sequencing data is shown on *top* in gray; *y*-axis is 1000 fpkm. TDP-43-CLIP sites are shown in the *bottom* track; each line represents a CLIP site (Hallegger et al. 2021). The direction of transcription is denoted by the arrows on the refseq genes. Exons are shown as closed blocks and introns as lines.

To directly compare the efficacy of ADAR (TRIBE) with that of APOBEC (STAMP) in HEK-293 cells, we took advantage of two existing TDP-43-ADAR TRIBE constructs (Herzog et al. 2020): the cytomegalovirus (CMV) promoter expressed either the TDP-43 coding sequence (cds) followed by the ADARcd or the ADARcd alone (pCMV-TDP-43-ADARcd and pCMV-ADARcd). Parallel constructs were generated in which the ADARcd was replaced with rat APOBEC1 as used in STAMP experiments (Fig. 1A; Brannan et al. 2021; pCMV-TDP-43-APOBEC and pCMV-APOBEC). These plasmids were transfected into HEK-293 cells along with a pCMV-eGFP plasmid. As a control, HEK cells were transformed with pCMV-EGFP only. Western blot analysis revealed that similar levels of TDP-43 fusion proteins were generated for TRIBE and STAMP, although TDP-43-APOBEC had slightly higher protein levels (Supplemental Fig. 1). Twenty-four hours after transfection, GFP-positive transfected cells were isolated using a Melody Fluorescence Activated Cell sorter (BD Melody FACS). RNA sequencing libraries were generated from two different biological replicates using Smart-seq2 (see Materials and Methods).

Existing TRIBE computational pipelines were used to identify both A to G editing events (TDP-43-ADAR and ADAR-only) as well as C to T editing events (TDP-43-APOBEC and APOBEC-only) in the RNA sequencing data (McMahon et al. 2016; Rahman et al. 2018; see Materials and Methods). In short, libraries were trimmed and mapped to the human genome (GRChg38.p13). Differential gene expression analysis was performed to verify that expression of TDP-43-ADAR and TDP-43-APOBEC did not cause dramatic changes to the transcriptomes of the HEK cells (Supplemental Fig. 2). To identify editing sites, the numbers of A, G, C, and T at every position in the genome were uploaded to a MySQL database. To be considered an editing site, a particular genomic location needed to be encoded by predominantly the nonedited base (adenosine [ADAR] or cytosine [APOBEC]) in the HEK cells expressing only EGFP (see Materials and Methods). If this criterion was met, the same genomic location was examined in the experimental samples. To score as edited, a location required at least 20 reads and >6% A to G editing (ADAR) or C to T editing (APOBEC; see Materials and Methods).

Expression of TDP-43-ADAR and TDP-43-APOBEC in HEK cells resulted in 35,000–45,000 editing sites in both biological replicates of the sequencing libraries (Fig. 1B). The TDP-43-editing enzyme fusions generated substantially more editing than expressing the editing enzymes alone (in gray), showing that RNA editing events are substantially increased by fusing these enzymes to an RBP. As a preliminary investigation of editing site location, we examined these sites on three TDP-43 target transcripts: a known target of TDP-43 (TDP-43 mRNA itself; the gene encoding this mRNA is TARDBP; Ayala et al. 2011; Polymenidou et al. 2011) as well as two putative targets, NET1 and CD59 (Fig. 1C). TDP-43-ADAR (red; two biological replicates) and TDP-43-APOBEC (blue; two biological replicates) edit similar mRNA regions of all three genes. These editing clusters are often colocalized with putative TDP-43 binding sites discovered by CLIP (bottom panel Fig. 1C; lines indicate known CLIP target sites; Hallegger et al. 2021). Expression of the editing enzymes alone (APOBEC, green; ADAR, orange; two biological replicates each) resulted in many fewer editing sites on these target mRNAs (Fig. 1B,C).

**FIGURE 2.**
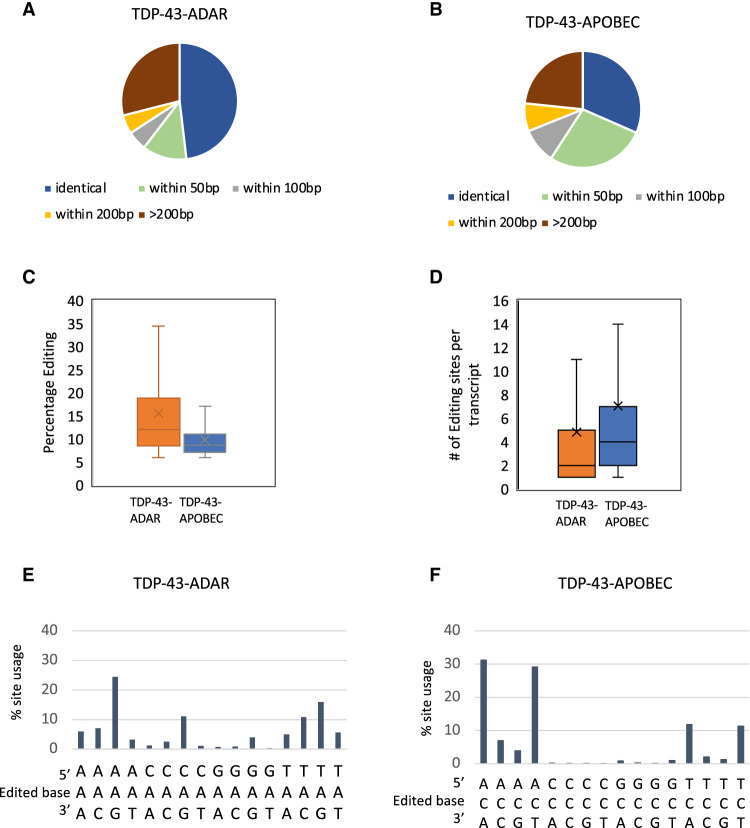
TDP-43 TRIBE and TDP-43 STAMP identify editing sites with different characteristics on TDP-43 target transcripts. (*A*,*B*) Pie charts showing the relationship between editing sites in two biological replicates. Editing sites identified in both experiments at: identical locations (dark blue), two different locations that are in close proximity in the biological replicates (green and gray; 50–100 bp), within 200 bp (yellow), or >200 bp (dark red). (*C*) Mean percentage editing for all editing sites identified in TDP-43-ADAR (∼15%) and TDP-43-APOBEC (∼10%). Mean values indicated by *X* and median values by lines (*P*-value <0.0001; Wilcoxon rank-sum test). (*D*) Mean editing sites per transcript generated by TDP-43-APOBEC (∼7) and TDP-43-ADAR (∼5) (mean indicated by *X* and median indicated by a line; *P*-value <0.0001; Wilcoxon rank-sum test). (*E*,*F*) The near neighbors of all edited nucleotides were identified and quantified (percentage of all editing sites). TDP-43-ADAR (*E*) preferentially edited adenosines followed by guanosine. TDP-43-APOBEC (*F*) preferentially edited cytosines that were flanked by A or T.

Although the number of RNA editing sites generated by TDP-43-ADAR and TDP-43-APOBEC were quite similar, the two enzymes show different editing characteristics. First, expressing APOBEC alone generated fivefold higher levels of editing than expressing ADARcd alone (Fig. 1B). This may be because the entire APOBEC coding sequence was used compared to only the catalytic domain of ADAR (see Discussion). Second, ADAR more often edits the exact same nucleotides in two biological replicates (48% of sites are identical; another 17% are within 100 bp), whereas APOBEC is more likely to edit nearby nucleotides (only 31% of sites are identical and 37% are within 100 bp; compare Fig. 2A,B). Third, ADAR generates an overall higher level of editing at each site (Fig. 2C). The average percentage editing of a TDP-43-ADAR site is 15.5% compared to 9.8% for TDP-43-APOBEC. This is undoubtedly related to the observation that ADAR often edits the same nucleotide in replicate experiments, that is, RBP–ADAR is more likely to edit the same A in another copy of the same transcript rather than nearby locations, which results in more reproducibility and a higher editing percentage. Fourth, TDP-43-ADAR yields fewer edited nucleotides per mRNA (mean of 4.8) compared to TDP-43-APOBEC (mean of 7; Fig. 2D). This observation is also because TDP-43-ADAR is more likely to edit the same nucleotide, which yields higher editing percentages at this location but fewer overall edited nucleotides on a target mRNA.

We hypothesized that this more localized preference of ADAR may be because it has more restricted site choice rules than APOBEC. To compare these rules, we computationally extracted the neighboring nucleotide 5′-and 3′ of the edited sites (Fig. 2E,F). Surprisingly, TDP-43-ADAR had a less restricted site choice than TDP-43-APOBEC (Fig. 2E,F). Eighty-four percent of TDP-43-APOBEC editing events occurred on a C flanked by only A or T (Fig. 2F; ACA, ACT, TCT, and TCA). Editing sites were rarely observed on cytosines with a 5′-C or 5′-G. TDP-43-ADAR also showed specific site choice but to a lesser extent: 64% of the TDP-43-ADAR editing sites were concentrated on AAG, CAG, TAC, and TAG (Fig. 2E). These observations are consistent with previous work showing that the ADAR2-E488Q catalytic domain prefers T or A 5′-of the editing sites (Kuttan and Bass 2012). The higher restrictive site choice of APOBEC suggests that the localized preferences of ADAR is due to RNA secondary structure (see Discussion).

**FIGURE 3.**
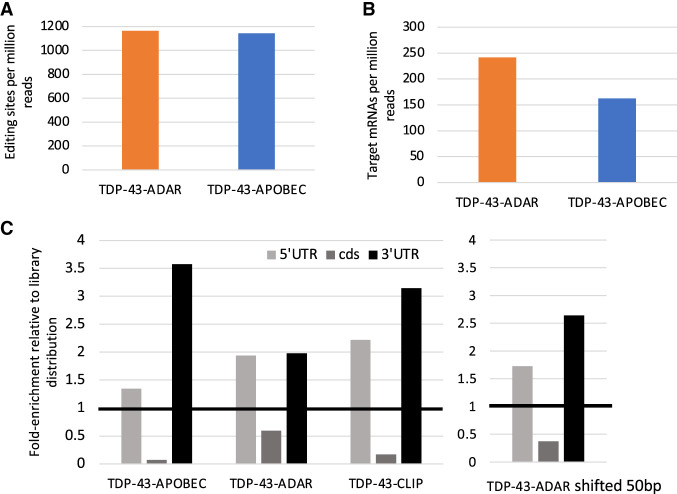
TDP-43-ADAR and TDP-43-APOBEC generated similar numbers of editing sites that were preferentially located in the 3′-UTR and 5′-UTR. (*A*) Quantification of the number of editing sites generated by TDP-43-ADAR and TDP-43-APOBEC (A to G changes are indicated for ADAR and C to T changes for APOBEC). This graph shows only editing sites that were consistent between two biological replicates not found in enzyme-only controls. The number of editing sites identified was normalized per million reads to adjust for sequencing library depth. (*B*) The number of target transcripts identified by TDP-43-ADAR and TDP-43-APOBEC. (*C*) The distribution of editing sites in the 5′-UTR, coding sequence (cds), and 3′-UTR was calculated for TDP-43-ADAR and TDP-43-APOBEC as well as TDP-43-CLIP. The graphs show the fold enrichment relative to the read distribution of the RNA sequencing library. In all cases, the 3′-UTR and 5′-UTR enrichment is significant using a proportion test; *P*-value <0.0001. CLIP data is from Hallegger et al. (2021). (*Right*) TDP-43-ADAR editing sites were shifted by 50 bp toward the end of the transcripts and the localization of the sites was reexamined. There was a 16% increase in the number of sites in the 3′-UTR.

To accommodate these differences in editing between TDP-43-ADAR and TDP-43-APOBEC and to make a pipeline useful for both enzymes, we made several modifications to our previous TRIBE bioinformatics pipeline. To account for the lower reproducibility of the APOBEC editing sites, we required an APOBEC editing site to be within 100 bp of a second site in the biological replicate instead of requiring the same site to be identified in two biological replicates as done for ADAR. In addition, we ignored any nucleotide edited at a frequency above 1% in either replicate of the enzyme-only samples. This conservative filtering step accommodates the higher background editing by the APOBEC-only construct and has been previously used in mammalian cells to ensure that editing sites identified with fusion proteins are not false positives (Biswas et al. 2020). After incorporating these two additional filtering steps, a final set of editing sites and target mRNAs were identified for TDP-43-ADAR and TDP-43-APOBEC (Fig. 3A). Although a nearly identical number of editing sites were identified for the two fusion proteins, TDP-43-APOBEC generated more sites per mRNA and therefore fewer target transcripts (Fig. 3B).

CLIP experiments indicate that TDP-43 binds to introns as well as to the 3′-UTRs of target transcripts (Polymenidou et al. 2011; Hallegger et al. 2021). Since the Smart-seq libraries generated in this study are generated from poly(A) mRNA, introns should and do represent a very small portion of sequenced transcripts. Editing sites were however enriched in the 3′-UTRs of target transcripts. This was shown by mapping TDP-43-ADAR or TDP-43-APOBEC editing sites as well as TDP-43 CLIP binding sites (Hallegger et al. 2021) to the genome and calculating the percentage of sites within the 5′-UTR, cds, and 3′-UTR. This distribution was compared to the transcriptome distribution and graphed as a fold change relative to that distribution (Fig. 3C).

All three samples show strong enrichment in 5′-UTRs as well as 3′-UTRs with an underrepresentation of sites in the cds. A higher percentage of TDP-43-ADAR editing sites map to the cds than those from TDP-43-APOBEC and TDP-43-CLIP. However, if the coordinates of the TDP-43-ADAR editing site are expanded by 50 bp, 16% of the TDP-43-ADAR editing sites shift from being the cds to the 3′-UTR (Fig. 3C; right). It is therefore possible that many of these binding events occur in the 3′-UTR near the cds-3′-UTR border.

**FIGURE 4.**
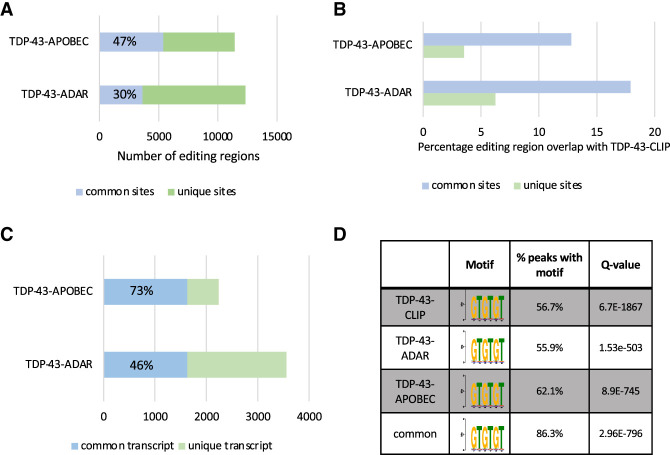
TDP-43-ADAR and TDP-43-APOBEC identified common edited regions and transcripts. (*A*) The coordinates of identified editing sites found in TDP-43-ADAR and TDP-43-APOBEC was expanded by 100 bp in each direction and examined for overlapping regions (>1 bp was considered overlapping). Edited regions found in both TDP-43 TRIBE and TDP-43 STAMP are indicated in blue, and those editing regions that were unique to either TDP-43-ADAR or TDP-43-APOBEC are shown in green. (*B*) The editing sites identified by TDP-43-APOBEC and TDP-43-ADAR were split into two groups: common sites (those found by both TDP-43-ADAR and TDP-43-APOBEC; blue) or unique sites (found only by one of the editing enzymes; green). These two subsets of editing sites were then overlapped with TDP-43 CLIP sites (any overlap >1 bp was considered overlapping). The percentage of the editing sites overlapping regions containing CLIP peaks is shown. (*C*) The transcripts identified by TDP-43-ADAR and TDP-43-APOBEC were compared. The graph indicates the percentage of transcripts that are found by both TRIBE and STAMP (blue) or were unique to either TRIBE or STAMP (green). (*D*) The 100 bp region surrounding RNA editing sites generated by TDP-43-ADAR, TDP-43-APOBEC, and TDP-43-CLIP sites was analyzed for binding motifs using Xstreme (Grant and Bailey 2021). The table indicates the percentage of edited regions that contain GU/GT-rich motifs with high significance.

The 3′-UTR and 5′-UTR enrichment of TDP-43-ADAR, TDP-43-APOBEC, and TDP-43-CLIP sites are quite similar. To determine whether these three methods identify the same binding regions, we expanded each editing or CLIP site by 100 bp in both directions and then compared the three regions (Fig. 4A,B). Forty-seven percent of the TDP-43-APOBEC and 30% of the TDP-43-ADAR sites are in the same genes and overlapping (Fig. 4A; blue), whereas the other edited regions were unique to TDP-43-APOBEC and TDP-43-ADAR (Fig. 4A; green). We then asked whether editing sites that are present in both TDP-43-ADAR and TDP-43-APOBEC (common sites) are more likely to also be identified by CLIP than sites found by either TDP-43 TRIBE or STAMP (unique sites). Indeed, there is a three- to fourfold increase in the percentage of common sites that are also identified in CLIP experiments (blue; Fig. 4B).

Because RBP–ADAR fusions often edit nucleotides as far as 500 nt from the RBP binding site (Xu et al. 2018), TDP-43-ADAR and TDP-43-APOBEC may identify similar transcripts even if their editing sites are not within 200 bp of one another. We therefore compared the lists of mRNAs identified as TDP-43 targets by TDP-43-ADAR and TDP-43-APOBEC: 73% of the TDP-43-APOBEC transcripts and 46% of the TDP-43-ADAR transcripts are identified by both methods (Fig. 4C; blue).

Because TDP-43-ADAR and TDP-43-APOBEC identify a substantially overlapping set of transcripts, the two methods can be combined to identify a higher confidence set of TDP-43 targets; this is also suggested by the increased overlap between CLIP and the common edited regions. We therefore examined the 100 bp regions surrounding TDP-43-ADAR, TDP-43-APOBEC, and TDP-43-CLIP sites for the GU-rich motif that is associated with TDP-43-binding (Polymenidou et al. 2011; Tollervey et al. 2011). Since the GUGUGU or GTGTGT motif was originally identified in CLIP experiments, we first examined the 100 bp region surrounding TDP-43-CLIP sites. Fifty-seven percent of these sites contain a GTGTGT motif, a highly significant enrichment (Fig. 4D; *q* = 6.7 × 10^−1867^). The TDP-43-ADAR and TDP-43-APOBEC identified regions were also significantly enriched for GTGTGT motifs; a similar percentage of these regions, 56% and 62%, contain GTGTGT motifs (Fig. 4D). For regions identified by both methods, the GTGTGT motif was identified in 86% of the binding regions with even higher statistical significance (Fig. 4D). This suggests that higher confidence binding regions can be identified by combining TRIBE and STAMP.

### Comparison of TRIBE and STAMP in *Drosophila*

**FIGURE 5.**
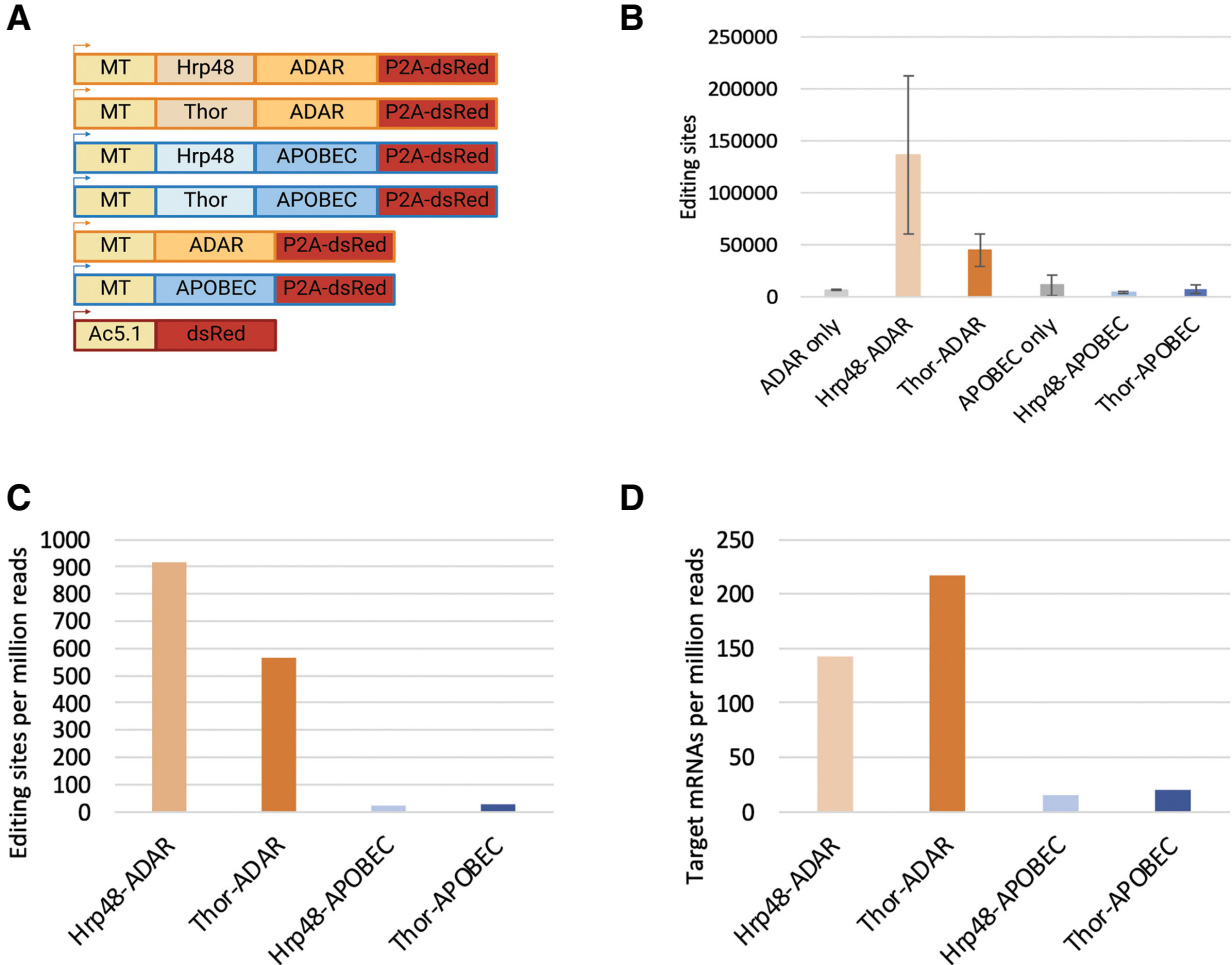
STAMP does not work well in *Drosophila* S2 cells. (*A*) Schematic of the expression constructs used to test TRIBE and STAMP in *Drosophila* S2 cells. (*B*) The average number of editing sites identified using Hrp48 and Thor TRIBE (orange) and STAMP (blue) in two biological replicates. ADAR and APOBEC-only controls are also shown to illustrate the background editing of each enzyme (gray). Error bars indicate standard deviation. (*C*) Editing sites identified in both biological replicates and not found in enzyme-only controls were quantified and normalized relative to the total number of reads in the RNA sequencing library. (*D*) The number of target transcripts identified by applying TRIBE and STAMP fused to Hrp48 and Thor are indicated.

We decided to follow the success of combining the ADAR and APOBEC editing enzymes to identify higher confidence TDP-43 targets in mammalian cells by testing the two enzymes side-by-side in *Drosophila* Schneider 2 cells (*Drosophila* S2 cells). To this end, we adopted the same strategy used above in mammalian cells and replaced the ADARcd with the rat APOBEC1 cds. TRIBE was originally developed in *Drosophila*, and we assayed the same two RBPs previously used to identify TRIBE targets in S2 cells, namely, Hrp48 and Thor (*Drosophila* EIF4E-BP; McMahon et al. 2016; Jin et al. 2020). This resulted in a panel of plasmids that contained the metallothionein inducible promoter (MT) driving an RBP-editing enzyme fusion followed by a p2A self-cleaving peptide and dsRed for visualization (Fig. 5A). We also generated enzyme-only and dsRed only controls using the same general strategy. We transfected these expression plasmids into *Drosophila* S2 cells and isolated dsRed-positive cells via FACS (BD Melody). Poly(A) mRNA was isolated from 400 cells and was used as input for RNA sequencing libraries generated using Smart-seq2 (Picelli et al. 2014). RNA editing sites were identified using the new TRIBE computational pipeline described above adapted for *Drosophila* (see Materials and Methods).

As previously shown, expression of both Hrp48-ADAR and Thor-ADAR dramatically increased the number of editing sites above the low level of editing achieved by expressing the ADARcd alone (Fig. 5B; orange and light gray; McMahon et al. 2016; Jin et al. 2020). In contrast and unlike in human cells (Fig. 1B), expression of Hrp48-APOBEC and Thor-APOBEC did not cause an increase in editing above enzyme-only levels (Fig. 4B; blue and dark gray). This was not due to the instability of the APOBEC fusion proteins; western blotting could easily detect Hrp48-APOBEC (Supplemental Fig. 3). Moreover, the few C to T transitions appear due to APOBEC; these editing events are much more prevalent than other nucleotide changes and show the characteristic site choice of this enzyme (flanking A or T nucleotides; Supplemental Fig. 4). This suggests that both RBP–APOBEC fusions only inefficiently edit target mRNAs in *Drosophila* tissue culture cells. Indeed, the number of RBP–APOBEC editing sites and mRNA targets that passed threshold (consistent between the two biological replicates and not edited at >1% editing in the enzyme-only controls) was more than 10-fold less than the same RBPs fused to ADAR (Fig. 5C,D).

The experiments in human cells shown above indicated that RBP–APOBEC fusions were likely to edit more sites but with a lower percentage of editing (Fig. 2C). This same trend is observed in *Drosophila*; the mean editing percentage on Hrp48-APOBEC editing sites is 8.8%, while the mean editing of Hrp48-ADAR sites is significantly higher (26.1%; Supplemental Fig. 5). To test the possibility that requiring 6% editing was preventing the identification of RBP–APOBEC editing sites, we reanalyzed the data with a 4% editing cutoff (Supplemental Fig. 6A). Although the number of editing sites identified by Thor-APOBEC and Hrp48-APOBEC increased approximately threefold, the number of APOBEC-only editing sites increased 15-fold. This suggests that a too high editing threshold is not the reason for the weak RBP–APOBEC editing relative to APOBEC-only editing.

Another possible explanation for the lack of APOBEC editing in *Drosophila* S2 cells was proposed by a recent paper posted on bioRxiv (Doll et al. 2022). It indicates that APOBEC deaminase activity is poor at temperatures commonly used for S2 cells (18°–24°) but functional at 29°C; to test this possibility, we repeated the Hrp48-Apobec experiment in S2 cells grown at 28°C. Raising the temperature did not increase APOBEC editing activity when fused to Hrp48 in *Drosophila* cells; on the contrary, overall editing levels by APOBEC alone and by Hrp48-APOBEC were further reduced at 28°C (Supplemental Fig. 6B). These results suggest that STAMP may not be a viable option for detecting RBP target mRNAs in nonmammalian systems (see Discussion).

## DISCUSSION

RNA binding proteins guide RNAs throughout their life, by binding to nascent RNAs as they emerge from the polymerase, by facilitating the removal of introns and nuclear export, and then by modulating mRNA turnover, localization, and translation in the cytoplasm (Babitzke et al. 2009; Hocine et al. 2010; Das et al. 2021). Mutations in over 1000 RBPs are linked to human diseases including Fragile X and ALS (for review, see Gebauer et al. 2021). Therefore, it is critical to have reliable tools to identify the mRNA targets of RBPs, in specific cell types and even single cells. To this end, we directly compared in this manuscript TRIBE (RBP–ADAR) and STAMP (RBP–APOBEC) in both human cells and in *Drosophila* cells. In human cells, TDP-43 targets were successfully identified using both methods, and ∼70% of the STAMP targets were identified by TRIBE. The results also indicated that a higher confidence set of RBP targets is identified by defining the common targets identified with both methods.

Consistent with our finding that TRIBE and STAMP both work well and similarly is a very recent study that utilized both TRIBE and STAMP simultaneously, which the authors dubbed TRIBE-STAMP (Flamand et al. 2022). In this study, Flamand et al. investigated the mRNA targets of the m^6^A reader proteins, YTHDF1, YTHDF2, and YTHDF3 and showed that these three proteins identified similar target mRNAs with both TRIBE and STAMP. Although the overlap between these two methods was greater than observed here, this may be because the m^6^A reader proteins bound a very large percentage of the transcriptome, perhaps generating a higher likelihood of common targets. These authors also showed that TRIBE and STAMP can be combined to determine if two RPBs bind to the same transcript, another application of using both TRIBE and STAMP in parallel.

Although we were hopeful STAMP would also become a second tool for use in *Drosophila*, we were unable to observe significant RNA editing by APOBEC fused to two different RBPs in *Drosophila* S2 cells. The *Drosophila* constructs mirror those successfully used in human cells and in the original STAMP study, that is, APOBEC is fused to the carboxyl terminus of the RBP. The STAMP fusion proteins are transcribed and translated, and some APOBEC-derived editing is still detected (Fig. 5B; Supplemental Fig. 3). However, the Thor-APOBEC and Hrp48-APOBEC editing levels are similar to those observed in the APOBEC-only controls (Fig. 5B). This poor editing is not due to reduced enzyme activity at lower temperatures; similar results were obtained at 28°C (Supplemental Fig. 6B). These results indicate that STAMP is not a good choice for RBP target identification in *Drosophila* and perhaps in other invertebrate systems.

Does either TRIBE or STAMP pose a significant advantage for RBP target identification in mammalian cells? Does one method identify substantially more false negatives or false positives than the other?

As far as false negatives are concerned, each enzyme has intrinsic features that bias the editing of an RBP-bound RNA. TDP-43-APOBEC has a strong nearest neighbor preference as nearly all cytosines edited are flanked by A or T (84%; Fig. 2F). A previous study argued that APOBEC is ideal for RBP target mRNA identification. This is because it can edit cytosines in single-stranded mRNA, which should be between 25% and 35% of nucleotides in mammalian transcripts (Brannan et al. 2021). However, the strong nearest neighbor preference observed both here and in a previous study (Rosenberg et al. 2011) suggests that RBP–APOBEC fusion proteins edit a more limited set of cytosines. Nonetheless, the observation that TDP-43-APOBEC tends to edit different cytosines on the same transcript or multiple copies of the same transcript suggests that this nearest neighbor preference is not an issue for the identification of most mRNA targets and target regions.

In contrast, TDP-43-ADAR is more likely to edit the same adenosine resulting more often in the editing of the exact same nucleotide in multiple experiments (Fig. 2A). Since the ADARcd has less stringent nearest neighbor preferences than APOBEC (Fig. 2E,F), the repeat editing of specific adenosines by the ADARcd is more likely due to its double-stranded RNA requirement (Macbeth et al. 2005) and may be responsible for the fewer TRIBE edits compared to STAMP edits: an average of 4.8 sites/transcript for the ADARcd versus 7 sites/transcript for APOBEC (Fig. 2D). Despite this modest difference, TRIBE identifies somewhat more target transcripts than STAMP despite being expressed at lower levels (Fig. 3B; Supplemental Fig. 1). This indicates that the ADARcd double-stranded RNA requirement is not an obstacle to target identification as previously discussed (McMahon et al. 2016) and that TRIBE does not suffer from a severe false negative problem relative to STAMP.

This previous study has also purported that TRIBE is not well suited for RBP target identification compared to STAMP because the ADARcd only generates very few edits, only one to two per target transcript, and that TRIBE is incapable of editing coding regions (cds) due to their single-stranded nature (Brannan et al. 2021). This comment was based on results with Fmr1 using the first version of TRIBE prior to the adoption in 2018 of an ADARcd containing the E488Q mutation (Xu et al. 2018). This HyperTRIBE method gives rise to much more editing, due to faster editing speed and less preference for an UAG neighboring sequence surrounding the editing site (Kuttan and Bass 2012). In fact, as shown here and elsewhere, TRIBE identifies target transcripts with editing sites containing from 1 to 43 nt and also edits cds (Fig. 2D; Cheng et al. 2021).

What about false positives, editing sites that do not reflect RBP mRNA binding? TRIBE only uses the ADAR catalytic domain (ADARcd), avoiding its RNA binding regions. Probably as a consequence, the ADARcd alone generates a low level of A to G editing, suggesting that most TDP-43-ADARcd editing sites and target transcripts are bona fide positives. In contrast, the catalytic and RNA binding regions of APOBEC are not defined, requiring use of the entire protein in STAMP. This likely explains the fivefold increase in editing by APOBEC-only compared to ADAR-only (Fig. 1B).

The ability of APOBEC to edit single-stranded DNA could also impact the level of detectable background editing. Indeed, rat APOBEC1 used here and in the original STAMP study has also been used successfully in combination with CRISPR for genome editing (Komor et al. 2016, 2017). Other studies have shown that APOBEC1 can drive off-target DNA editing and RNA editing even when fused to Cas9 (Grunewald et al. 2019; Jin et al. 2019; Kanca et al. 2019; McGrath et al. 2019). This observation was true even with transient transfection of the Cas9–Apobec fusions (McGrath et al. 2019). To date, genomic DNA has not been examined in STAMP, making it uncertain whether APOBEC-driven single-stranded DNA editing is contributing to false positive editing sites and transcripts. Although current computational approaches cannot distinguish DNA editing from RNA editing, we attempted to ameliorate this issue by eliminating from the final list of editing sites any nucleotide that has >1% editing in enzyme-only control samples.

Other possible sources of false positive editing sites and target transcripts are shared by TRIBE and STAMP. First, these methods currently rely on overexpression, which can cause RBPs to bind to and edit secondary, low efficiency targets as well as primary high efficiency targets. Second, there are false-positive sources of editing that are intrinsic to living cells, endogenous editing by ADAR and APOBEC as well as genomic variation in the form of sNPs. Our computational pipeline is set up to handle these issues and is also comprehensive for both methods. Unlike computational approaches that identify editing sites by comparing the experimental RNA sample to publicly available genomic sequences, we directly compare RNA sequences from TRIBE and STAMP samples to the same cell line expressing only GFP and therefore eliminate most of these false positive sources, i.e., sites that are edited by endogenous ADAR and APOBEC as well as sNPs are present in the control cell line (see Materials and Methods). A third source of false positives is technical error, whether from PCR during RNA sequencing library generation or from sequencing itself. To control for this, we require edited nucleotides to have 20 reads of coverage with greater than one edited read to ensure that a candidate altered nucleotide is genuine. Although this requirement requires additional sequencing depth to detect editing events in less abundant transcripts (at least 12 million uniquely mapped reads for each sample), it prevents the identification of an editing site due to a single A to G (ADAR) or C to T (APOBEC) change that could be due to technical error. Although our previous TRIBE analyses have been done with editing percentage cutoffs as low as 5% (Biswas et al. 2020; Herzog et al. 2020) and as high as 10% (McMahon et al. 2016; Xu et al. 2018), we used in this work an editing cutoff (6%) dictated by the lower STAMP editing percentages as well as the by similar parameters used in the previous STAMP study (Brannan et al. 2021). In summary, our pipeline is generally conservative and designed to reduce the contribution of false positives while maintaining bona fide targets.

The topics of false positives and false negatives warrant a return to considering the lack of overlap between TRIBE, STAMP, and CLIP (Fig. 4). There is unfortunately no ground truth; just like TRIBE and STAMP, CLIP is also prone to false positives and false negatives (for review, see Xu et al. 2022). One conservative approach is to identify a set of high-confidence targets by overlapping methods. A positive hit in more than one method will decrease the likelihood that it is a false positive. This should be possible in mammalian systems by performing TRIBE and STAMP and moving forward with common targets. This will be especially useful when CLIP is not easy to apply. In *Drosophila* and in other systems in which applying more than one method is not feasible, the use of multiple biological replicates is still helpful in identifying the most consistent and reproducible RBP targets.

## MATERIALS AND METHODS

### Plasmids

The details of human and *Drosophila* plasmid construction are below and listed in Supplemental Table 1. For all plasmids, we used either Q5 (NEB) or Ex Taq (TaKaRa) DNA polymerases to PCR amplify inserts. All inserts and vectors were gel purified using the Gel Extraction Kit (Qiagen). We inserted all fragments into vectors using either Gibson Assembly (NEB) or NEBuilder (NEB) unless otherwise noted. All plasmids were transformed into NEBalpha DH5 high efficiency competent cells. We verified candidate plasmids using colony PCR using primers listed in Supplemental Table 2 and REDTaq ReadyMix (Sigma-Aldrich). All plasmids were miniprepped (Qiagen) and sequenced with Plamidsaurus (https://www.plasmidsaurus.com).

#### HEK-293 expression vectors

Plasmids for expressing TDP-43-ADAR (pCMV-hADARcd-E488Q) as well as the ADAR-only control (pCMV-hADARcd-E488Q) were previously published (Herzog et al. 2020). To generate pCMV-APOBEC (pCR25), we amplified rat APOBEC1 from pMT-Hrp48-APOBEC-P2A-dsRed (pCR8; see below) with primers CR60 and CR61 and inserted it into pCMV-ADARcd-E488Q digested with EcoRI and KpnI. To clone pCMV-TDP-43-APOBEC (pCR27), we amplified APOBEC from pMT-Hrp48-APOBEC-P2A-dsRed (pCR8) with primers CR73 and CR74 and inserted it into pCMV-TDP-43-ADARcd-E488Q linearized using PCR with primers CR70 and CR71.

#### Drosophila plasmids

A *Drosophila* HyperTRIBE plasmid was generated by including a self-cleaving peptide, p2A, followed by dsRed downstream from the Hrp48-ADAR sequence (pMT-Hrp48-ADAR-E488Q-P2A-dsRed; pCR1). This plasmid and all related plasmids have a V5-tag 5′ of the editing enzyme. The plasmid pMT-Tyf-ADAR-E488Q-P2A-dsRed was digested with PmeI and NotI to liberate ADAR-E488Q-P2A-dsRed. This fragment was ligated into PmeI and Not1-digested pMT-Hrp48-ADAR-E488Q using T4 ligase (NEB). pMT-Hrp48-APOBEC-P2A-dsRed (pCR8) was made by amplifying rat APOBEC1 from pCMV-BE1 (Addgene #73019) using primers CR26 and CR27. Gibson Assembly was then used to insert APOBEC1 into pMT-Hrp48-ADAR-E488Q-P2A-dsRED (pCR1) digested with ApaI and NotI to remove ADAR-E488Q. The resulting plasmid was then cut with NotI to insert a linker (made by annealing primers CR29 and CR30) using Gibson Assembly. To clone the APOBEC-only control (pMT-APOBEC-p2A-dsRed; pCR10), we amplified APOBEC from pCMV-BE1 (Addgene #73019) using primers CR28 and CR27. Plasmid pMT-Hrp48-ADAR-P2A-dsRed (pCR1) was digested with ApaI and KpnI to liberate Hrp48-ADAR and APOBEC was inserted using Gibson Assembly. To clone an ADAR-only control (pMT-ADAR-P2A-dsRed; pCR12), we digested pMT_Hrp48_ADAR_E488Q_P2A_dsRed (pCR1) with NotI and KpnI to remove Hrp48. We blunted the resulting sticky ends using Klenow end-blunting (NEB) and recircularized the plasmid using T4 blunt-end ligation (NEB). To clone pMT-Thor-APOBEC-P2A-dsRed (pCR26), we amplified Thor from pMT-Thor-Linker-HyperTRIBE (Jin et al. 2020) with primers CR66 and CR67 and inserted it into pMT-Hrp48-Linker-APOBEC-P2A-dsRed (pCR8) digested with KpnI and NotI to remove Hrp48 using NEBuilder (NEB). To clone pMT-Thor-ADAR-P2A-dsRed (pCR30), we amplified Thor from pMT-Thor-Linker-HyperTRIBE (Jin et al. 2020) using primers CR80 and CR81 and inserted it into pMT-Hrp48-ADAR-E488Q-P2A-dsRed (pCR1) digested with KpnI and NotI to remove Hrp48. Insertion was done using NEBuilder.

### Cell culture

HEK293T cells (ATCC CRL-3216) were cultured at 37°C in Gibco DMEM, high glucose, GlutaMAX supplement, pyruvate (Thermo Fisher) with 10% Fetalgro synthetic FBS (RMBio, FGR-BBT) and 1% Penicillin–Streptomycin (Genesee Scientific). HEK cells were transiently cotransfected with pCMV-EGFP and the relevant plasmid of interest in a six well plate using the Lipofectamine3000 protocol (Thermo Fisher). The CMV promoter is constitutively expressed and 24 h after transfection, GFP-positive cells were collected with BD FACS Melody.

*Drosophila* S2 cells were cultured at 23°C or 28°C in Schneider's media with 10% Gibco HI-FBS (Thermo Fisher) and 1% Gibco Antibiotic-Antimycotic (Thermo Fisher). *Drosophila* S2 cells were transiently transfected using the Mirus TransIT-2020 transfection protocol (Mirus Bio) in a six well plate for ∼24 h. Cells were induced for 24 h by inducing the metallothionein promoter (pMT) using 0.5 mM copper sulfate (Sigma). DsRed-positive cells were collected with the BD FACS Melody.

### RNA library preparation

Four-hundred cells were sorted directly into aliquots of 100 µL lysis buffer (Invitrogen Dynabeads mRNA Direct Kit; Thermo Fisher). Poly(A)-plus RNA was isolated using Invitrogen Dynabeads mRNA Direct Kit (Thermo Fisher, 61012) and sequencing libraries were prepared following Smart-Seq2 optimized protocol (Picelli et al. 2014). cDNA was quantified with D5000HS TapeStation (Agilent) and final tagmented libraries were quantified on D1000HS TapeStation (Agilent). Libraries were sequenced on Illumina NextSeq500 with 75 cycle High Output Kit v2.5.

### TRIBE pipeline

To identify editing sites, the TRIBE pipeline was used as previously described (Rahman et al. 2018; https://github.com/rosbashlab/HyperTRIBE). Briefly, custom scripts were used to trim (Cutadapt; Martin 2011) and align (STAR; Dobin et al. 2013) reads to the appropriate genome (Human [GRChg38.p13] or *Drosophila* [dm6]). Mapped reads were used to generate gene expression data (see below) as well as bigwig files for visualization on the Integrated Genomics Viewer (IGV) (Robinson et al. 2011). The mapped reads were then converted to a matrix file listing the number of As, Gs, Cs, and Ts found in sequencing reads at each genomic coordinate. This data was loaded into a MySQL database for efficient querying. Editing sites were identified by identifying coordinates in the control samples (EGFP control for HEK-293 cells and the dsRed control for *Drosophila* S2 cells) that meet three criteria: (1) At least 80% of the reads were an A (ADAR) or a C (APOBEC), (2) <0.5% of the reads at that location were the edited base, i.e., a G (ADAR) or a T (APOBEC), and (3) there were at least nine reads covering the location. If these three conditions were met, then these coordinates were examined in the experimental samples. The coordinate is considered an editing site if: (1) there are at least 20 reads in the experimental sample, and (2) >6% of the reads have an edited base (G [ADAR] and T [APOBEC]) at that location (see Rahman et al. 2018 and custom scripts). Note that although our original TRIBE studies used a 10% editing cutoff (McMahon et al. 2016), more recent TRIBE studies have implemented a lower editing threshold of 5% (Biswas et al. 2020; Herzog et al. 2020). We opted for a 6% editing cutoff for these studies so that our analysis was consistent with the previous STAMP analysis (Brannan et al. 2021). Supplemental Table 3 illustrates the effect of each filter on the total number of editing sites identified. By comparing experimental samples to a control RNA sample from the same cell line rather than publicly available genomic sequences, the majority of sNPs and endogenous editing events are filtered out in the initial editing site identification step.

Once editing sites were identified, a final list of editing sites for each TRIBE-RBP and STAMP-RBP was generated by identifying editing sites consistent between two biological replicates for RBP–ADAR and within 100 bp of each other in two biological replicates RBP–APOBEC. Both approaches utilized bedtools intersect and bedtools closest (Quinlan 2014). Editing sites with at least 1% editing in the editing enzyme-only control samples were identified using the custom script (Threshold_editsites_20reads.py) with editing threshold set to 0.01. The list of all sites identified with >1% editing in either biological replicate of the enzyme-only control libraries was then subtracted from the list of putative editing sites identified in the experimental samples using bedtools.

The number of editing sites per transcript was determined using a custom script (summarize_results.pl) which generates a list of all transcripts, the number of editing sites in that transcript (Rahman et al. 2018). Quantification of the distribution of RNA sequencing reads in the human and *Drosophila* RNA libraries was performed with read_distribution.py script in RSeQC4.0.0 (Wang et al. 2012). The distribution of editing sites between the 5′-UTR, cds, and 3′-UTR was determined using bedtools and custom scripts and compared to the distribution of reads in the sequencing library. Near neighbor preference was determined using custom scripts. To examine the overlap of TDP-43-TRIBE, TDP-43-STAMP, and TDP-43-CLIP binding regions, the editing or CLIP site was expanded by 100 bp in both directions using bedtools slop. Then the overlap (>1 bp) between the different regions was determined using bedtools intersect. All custom scripts generated for this study are annotated and deposited at https://github.com/rosbashlab/Comparison-of-TRIBE-and-STAMP.

To examine TDP-43-TRIBE, TDP-43-STAMP, and TDP-43-CLIP sites for the presence of GU-rich motifs preferentially bound by TDP-43, the region surrounding the editing site or CLIP site was expanded by 50 bp using bedtools slop. The resulting bed file was used to retrieve sequence information for these regions to use as input for motif analysis using Xstreme (Grant and Bailey 2021).

### CLIP data analysis

CLIP data was downloaded from ArrayExpress with accession number E-MTAB-9436 (Hallegger et al. 2021). CLIP data from two biological replicates was analyzed using iCount at iMAPS (https://imaps.goodwright.com/history/). CLIP peaks identified in both biological replicates were identified using bedtools and used as a high-confidence set of putative TDP-43-CLIP sites.

### Gene expression analysis

Raw gene count values were generated using HTseq.scripts.count during initial mapping of RNA sequencing (Anders et al. 2015). Two replicates of either TDP-43-ADAR or TDP-43-APOBEC were compared to two replicates of the negative control cells (HEK-293 cells expressing only EGFP). Prior to differential expression analysis, data was filtered to remove transcripts that were expressed at lower levels (expressed at less than 5 FPKM reads in more than two of the four samples). Differential gene expression upon overexpression of TRIBE and STAMP RBPs was analyzed using EdgeR (Robinson et al. 2010). The resulting smearplots are shown.

### Western blot analysis

HEK cells transfected with pCMV-TDP-43-hADAR-E488Q, pCMV-3HA-hADAR-E488Q, pCMV-TDP-43-APOBEC, pCMV-APOBEC, and pCMV-EGFP were lysed in RIPA buffer (10 mM HEPES pH 7.5, 5 mM Tris pH 7.5, 50 mM KCl, 10% glycerol, 2 mM EDTA, 1% Triton X-100, 0.4% NP-40, and protease cOmplete Mini tablets [Roche]). After centrifugation (15,000 rpm at 4°C for 10 min), samples were boiled in Laemmli buffer and loaded on a 10% MOPS NuPage Bis-Tris gel (Invitrogen) with the Novex Sharp Pre-stained Protein Standard (Invitrogen).

S2 cells transfected with pMT-Hrp48-APOBEC-P2A-dsRed (pCR8) and pMT-APOBEC-P2A-dsRED (pCR10) in wells of a six well plate and induced with 0.5 mM copper sulfate for 24 h. Two-hundred and fifty microliters of RIPA buffer was added to each well; cells were scraped and lysed at 4°C for 15 min. After centrifugation (15,000 rpm at 4°C for 10 min), samples were boiled in Laemmli buffer and loaded into a 10% MES NuPage Bis-Tris gel (Invitrogen) with the Novex Sharp Pre-stained Protein Standard (Invitrogen).

The gels were transferred to nitrocellulose using iBLOT2 (Invitrogen) following manufacturer's protocol. The blots were blocked with 5% milk in Tris-buffered saline with 1% Tween 20 (TBST) for 1 h before being incubated overnight at 4°C in primary antibodies (1:1000 mouse anti-V5 Tag Monoclonal Antibody [Invitrogen], 1:1000 anti-Actin monoclonal antibody [Invitrogen], or 1:500 anti-TDP-43 [Proteintech] in 5% milk/TBST). Primary antibodies were removed with six washes of TBST each for 5 min. The blots were incubated with secondary antibodies (Cytiva Life Science anti-Mouse IgG, peroxidase-linked antibody from sheep [Fisher Scientific] or Cytiva Life Science anti-Rabbit IgG, peroxidase-linked antibody from donkey [Fisher Scientific]) diluted in 5% milk/TBST for 1 h on a rocker at room temperature. The blots were then washed 6× for 5 min in TBST prior to being developed with Clarity Western ECL Substrate Kit (Bio-Rad). To examine actin levels as a loading control blots were stripped using Restore Western Blot Stripping Buffer (Thermo Scientific) for 15 min and reblocked with 5% milk for 1 h at RT on rocker. Blots were imaged using a ChemiDoc (Bio-Rad).

## DATA DEPOSITION

All sequencing data generated in this study is deposited in the Gene Expression Omnibus (GEO) under accession number GSE223557. Human data is in subseries GSE223555 and *Drosophila* data is in subseries GSE223556. The TRIBE analysis pipeline is publicly available at https://github.com/rosbashlab/HyperTRIBE. All scripts used in this manuscript are available at https://github.com/rosbashlab/Comparison-of-TRIBE-and-STAMP.

## SUPPLEMENTAL MATERIAL

Supplemental material is available for this article.

## Supplementary Material

Supplemental Material
