# Supplementary material for: Comparison of TRIBE and STAMP for identifying targets of RNA binding proteins in human and Drosophila cells
Source: RNA. 2023 Aug;29(8):1230–42. doi: 10.1261/rna.079608.123 (PMC10351885; doi:10.1261/rna.079608.123)
Supplement: Supplemental Material [file supp_29_8_1230__DC1.html]

Comparison of TRIBE and STAMP for identifying targets of RNA binding proteins in human and Drosophila cells — Supplemental Material 

# Comparison of TRIBE and STAMP for identifying targets of RNA binding proteins in human and *Drosophila* cells

## Supplemental Material

- Supplemental\_Figure\_1.pdf
- Supplemental\_Figure\_2.pdf
- Supplemental\_Figure\_3.pdf
- Supplemental\_Figure\_4.pdf
- Supplemental\_Figure\_5.pdf
- Supplemental\_Figure\_6.pdf
- Supplemental\_Tables.xlsx
- Supplemental\_Data\_1\_.xlsx
- Supplemental\_Data\_2\_.xlsx
- Supplemental\_Legends.docx
