# Supplementary figures and images for: Comparison of TRIBE and STAMP for identifying targets of RNA binding proteins in human and Drosophila cells
Source: RNA. 2023 Aug;29(8):1230–42. doi: 10.1261/rna.079608.123 (PMC10351885; doi:10.1261/rna.079608.123)

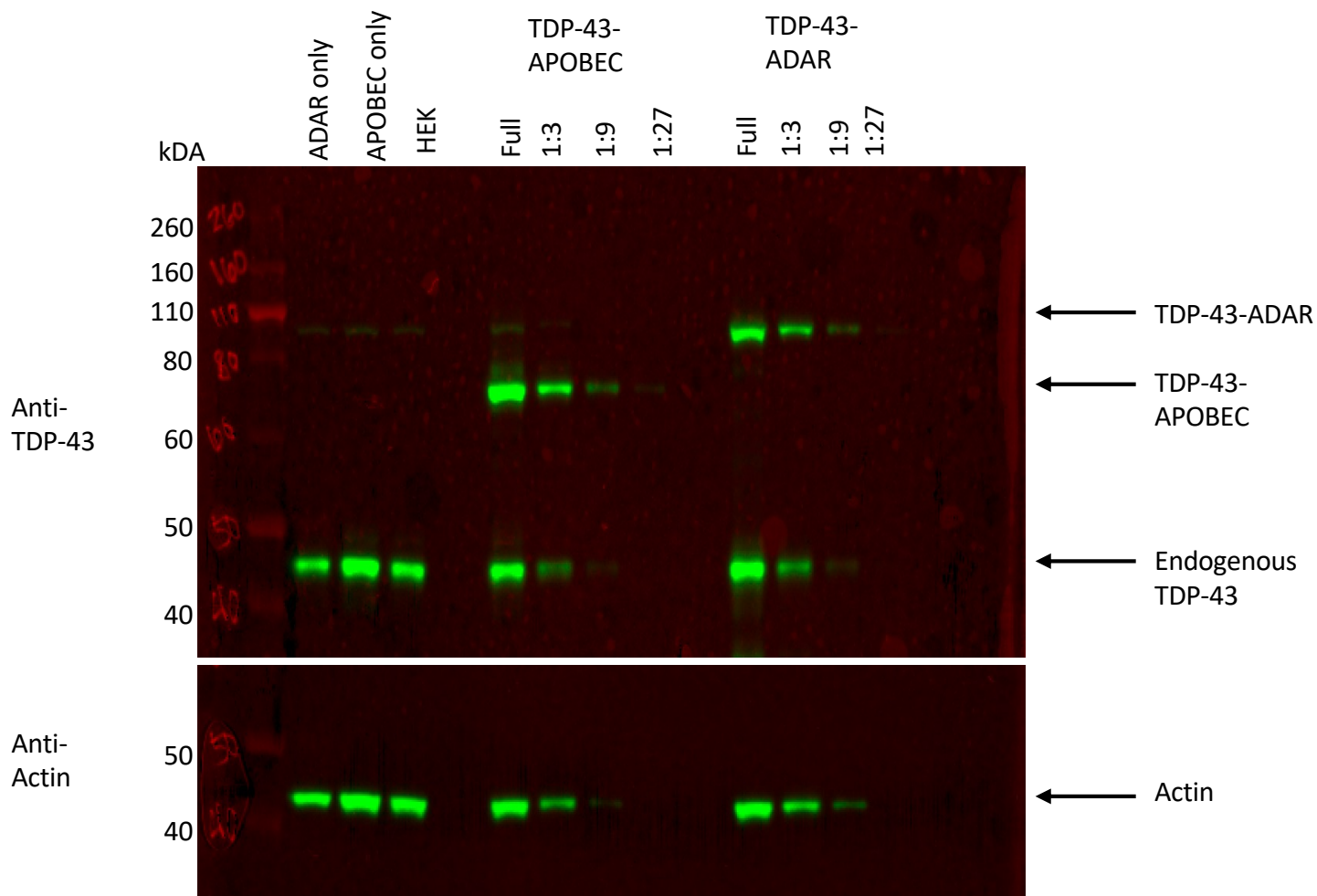

Supplemental Figure 1

Supplement: Supplemental Material [file supp_079608.123_Supplemental_Figure_1.pdf]

TDP-43-ADAR

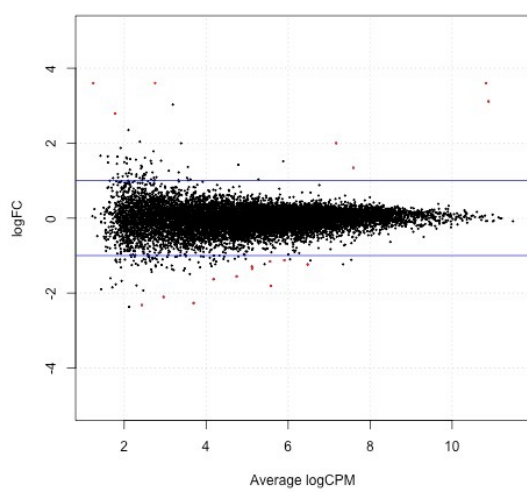

TDP-43-APOBEC

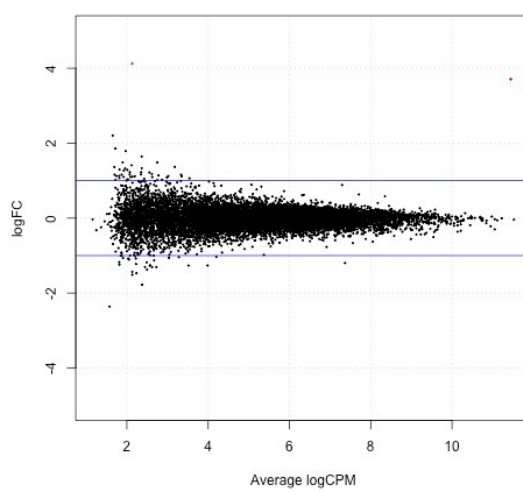

Supplement: Supplemental Material [file supp_079608.123_Supplemental_Figure_2.pdf]

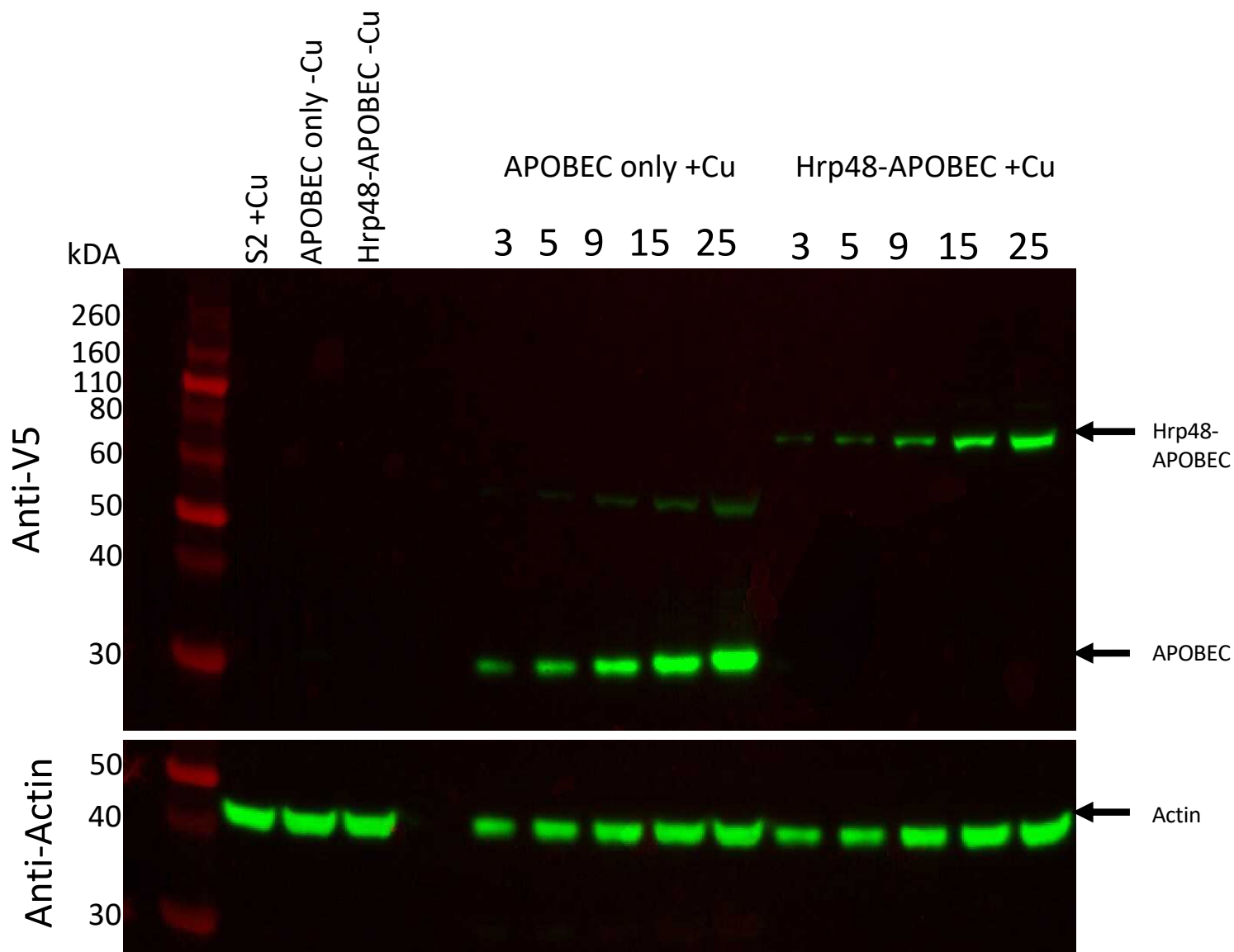

Supplemental Figure 3

Supplement: Supplemental Material [file supp_079608.123_Supplemental_Figure_3.pdf]

## Hrp48-APOBEC

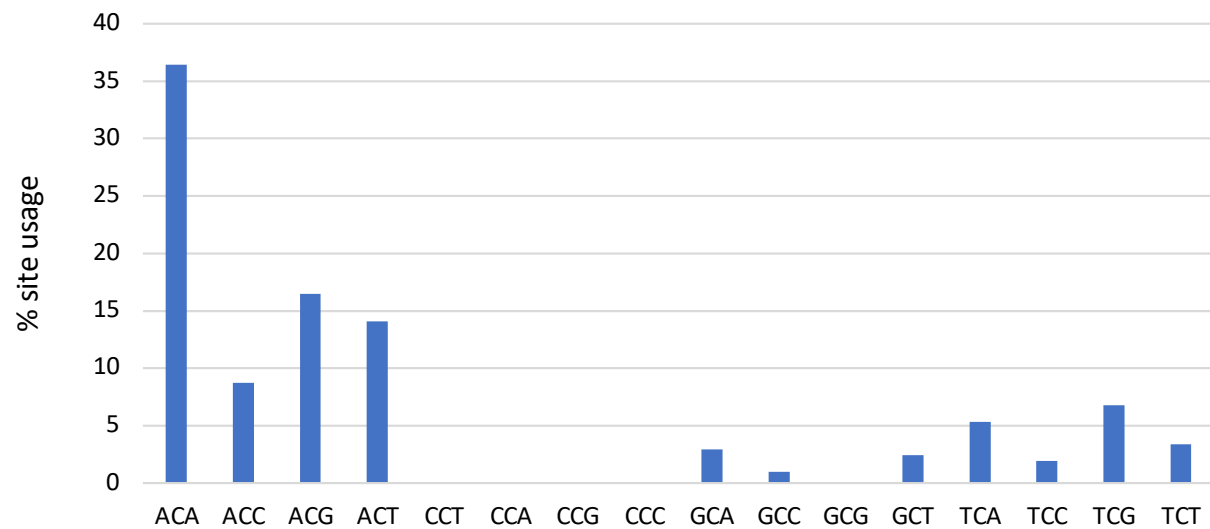

## Thor-APOBEC

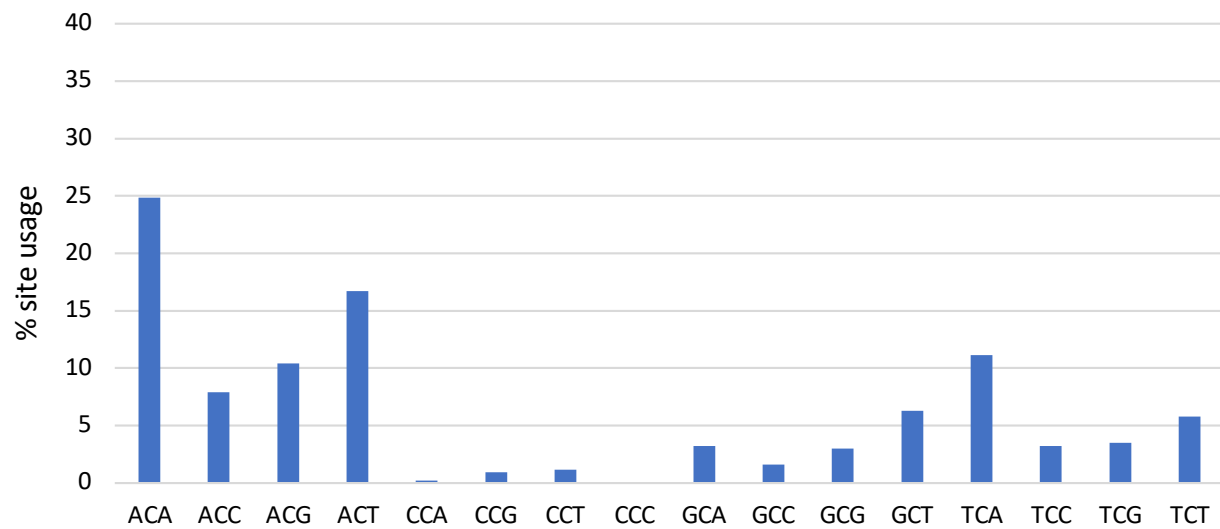

Supplement: Supplemental Material [file supp_079608.123_Supplemental_Figure_4.pdf]

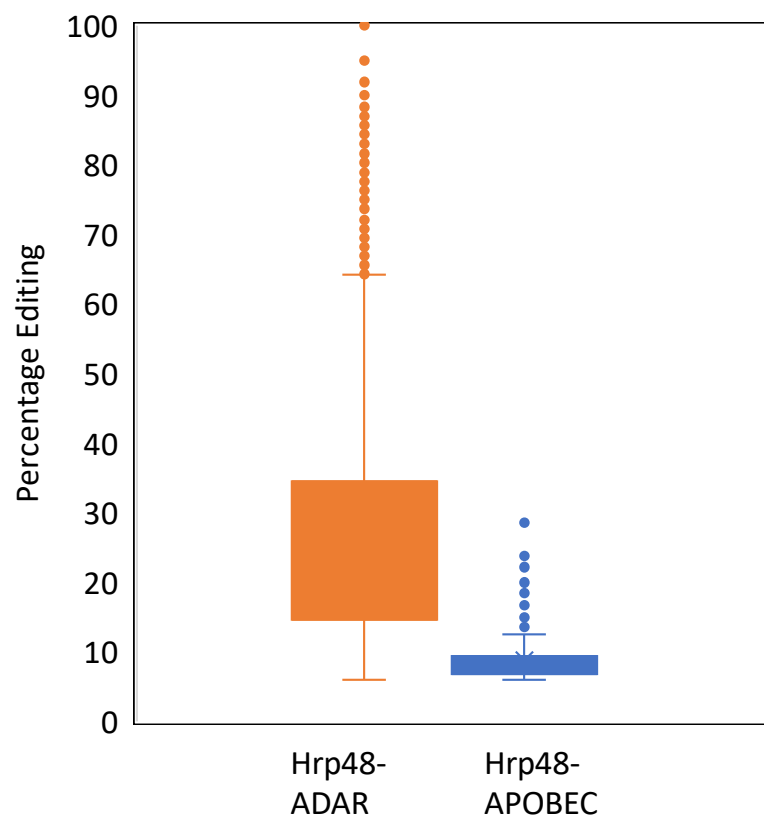

Supplemental Figure 5

Supplement: Supplemental Material [file supp_079608.123_Supplemental_Figure_5.pdf]

A)

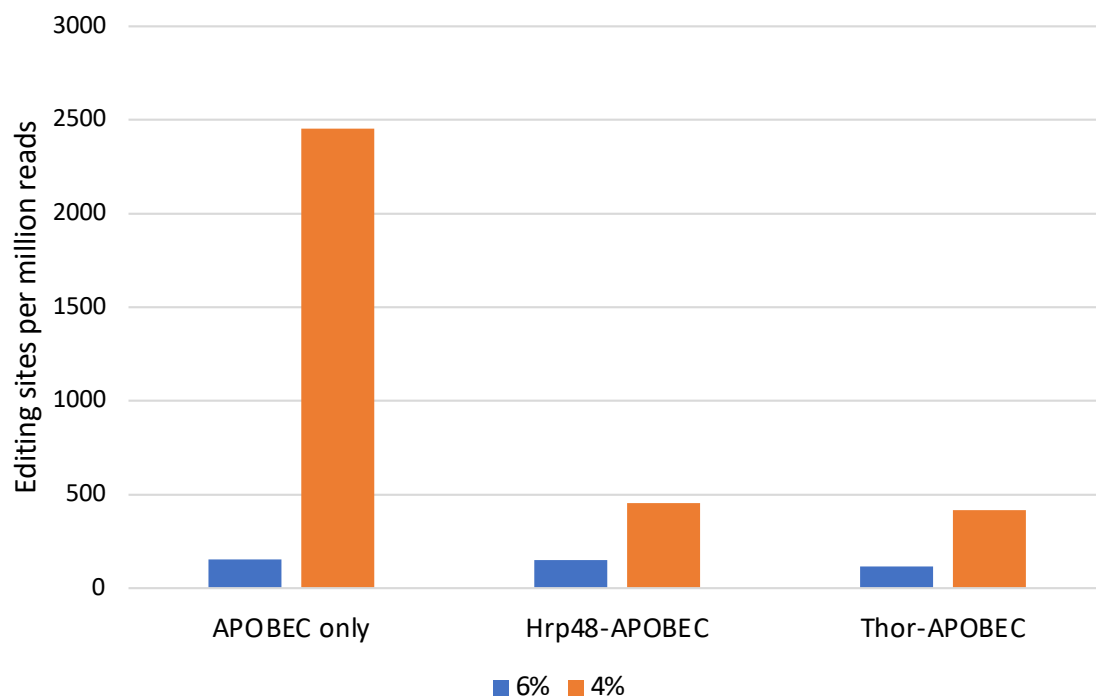

B)

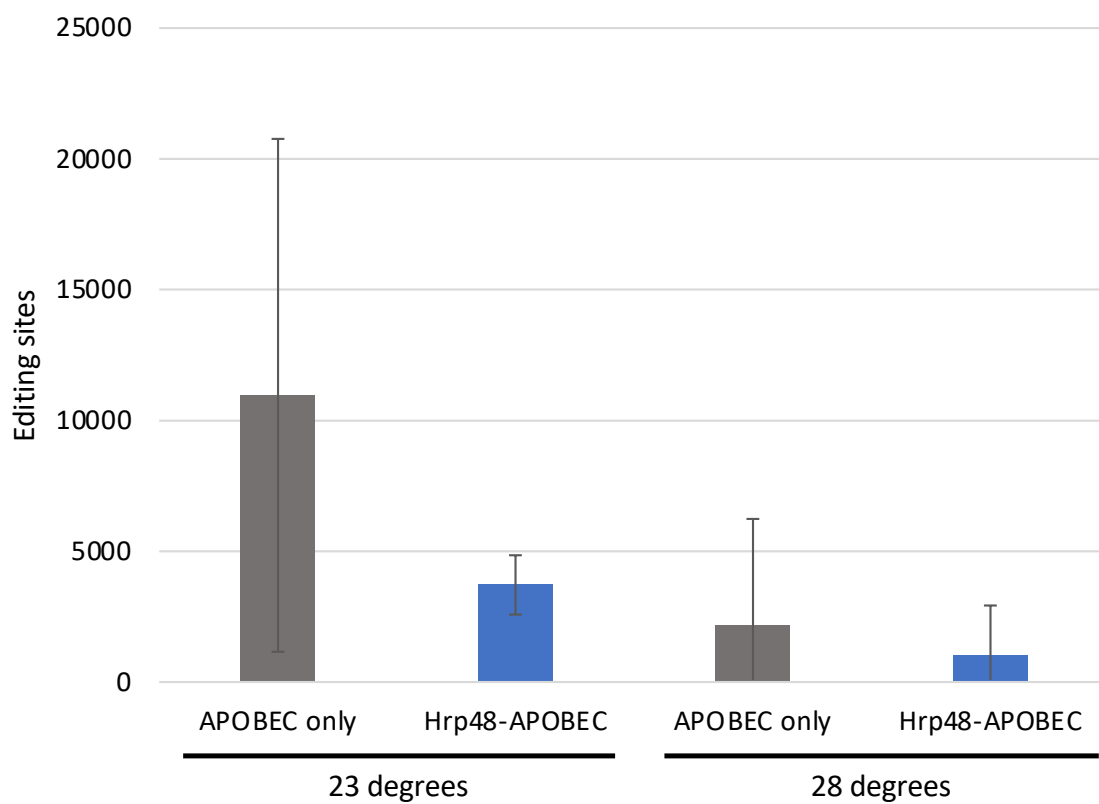

Supplemental Figure 6

Supplement: Supplemental Material [file supp_079608.123_Supplemental_Figure_6.pdf]
