# Supplementary material for: Comparison of TRIBE and STAMP for identifying targets of RNA binding proteins in human and Drosophila cells
Source: RNA. 2023 Aug;29(8):1230–42. doi: 10.1261/rna.079608.123 (PMC10351885; doi:10.1261/rna.079608.123)
Supplement: Supplemental Material [file supp_079608.123_Supplemental_Legends.docx]

**Supplemental Figure 1. TDP-43 protein levels in HEK-293 cells expressing TDP-43-ADAR, TDP-43-APOBEC, APOBEC only and ADAR only.** Anti-TDP-43 (TOP) and anti-Actin (bottom) western blots of protein extracts from HEK cells expressing ADAR only (lane 1), APOBEC only (lane 2), eGFP control plasmid (lane 3), TDP-43-APOBEC (lanes 5-8; dilution series), and TDP-43-ADAR (lanes 10-13; dilution series). The expected sizes of endogenous TDP-43, TDP-43-ADAR and TDP-43-APOBEC are indicated with arrows.

**Supplemental Figure 2. TDP-43-ADAR and TDP-43-APOBEC did not dramatically perturb HEK-293 cell transcriptomes.** Smear plots generated by EdgeR analysis comparing HEK cells expressing GFP to HEK cells expressing TDP-43-ADAR (left) or TDP-43-APOBEC (right). All transcripts expressed at greater than 5 FPKM in at least two experiments included in the analysis. Gray lines indicate a threshold for a 2-fold change. Significantly altered genes (q< 0.05) are indicated as red dots.

**Supplemental Figure 3. Hrp48-APOBEC and APOBEC proteins were expressed in *Drosophila* S2 cells.** Anti-V5 (TOP) and anti-Actin (bottom) western blots of protein extracts from *Drosophila* S2 cells expressing either APOBEC only or Hrp48-APOBEC under the control of the copper inducible metallothionein promoter. Wild-type *Drosophila* S2 cells induced with copper are shown in lane1 as a negative control. Additional negative controls are shown in lane 2 and 3: APOBEC only and Hrp48-APOBEC transfected cells without copper induction. Lanes 5-9 show increasing amounts of copper induced APOBEC only cell lysates (3µl to 25µL). Lanes 11-15 show increasing amounts of copper induced Hrp48-APOBEC cell lysates (3µl to 25µL). APOBEC and HRP48-APOBEC were both tagged with a V5 tag and western blotting shows bands at the expected sizes. Anti-actin blot (bottom) included as a loading control.

**Supplemental Figure 4. Editing sites generated by expression of Hrp48-APOBEC and Thor-APOBEC show bias toward cytosines flanked by A or T**. The near neighbors of all edited nucleotides were identified and quantified. The use of each near neighbor possibility is graphed as a % of all editing sites.

**Supplemental Figure 5. Editing sites identified in Hrp48-ADAR have a higher percentage editing than those sites identified in Hrp48-APOBEC.** The percentage editing at each editing site generated by TDP-43-ADAR (orange) and TDP-43-APOBEC (blue) was visualized using a box plot. Mean values indicated by X and median values by lines (p-value <0.0001; Wilcoxen-Rank Sum Test).

**Supplemental Figure 6: Lower numbers of editing sites generated by APOBEC in *Drosophila* S2 cells is not due to a lower editing percentage or low temperatures.** A) The number of editing sites identified by Hrp48-APOBEC, Thor-APOBEC and APOBEC alone is shown using a 6% editing cutoff (as used for all other analyses in this study; blue) and a 4% editing cutoff (orange). B) The number of editing sites identified in APOBEC only or Hrp48-APOBEC expressing *Drosophila* S2 cells grown at 23°C or 28°C.

**Supplemental Table 1: Plasmids used in this study.**

**Supplemental Table 2: Primers used in this study.**

**Supplemental Table 3: Editing site filtering.** Chart illustrating different steps in editing site identification and how many sites are lost at each filtering step.

**Supplemental Data 1: Editing sites and target transcripts identified by HEK-293 cells.** Excel worksheet with tables listing the editing sites and target genes identified in TDP-43-TRIBE and STAMP.

**Supplemental Data 2: Editing sites and target transcripts identified in *Drosophila* S2 cells*.*** Excel worksheet with tables listing the final editing sites and target genes identified in Hrp48 and Thor TRIBE and STAMP.
